# Some Curiosities of Medical Practice1An address delivered before the Buffalo Historical Society, May 17, 1903.

**Published:** 1903-08

**Authors:** Chauncey Pelton Smith

**Affiliations:** Buffalo, N. Y.


### BUFFALO MEDICAL JOURNAL.

VOL. XLIII.—LIX. AUGUST, 1903. NO. 1.

### ORIGINAL COMMUNICATIONS.

### Some Curiosities of Medical Practice.[1]

BY CHAUNCEY PELTON SMITH, M. D., Buffalo, N. Y.

THIS society has had the pleasure of listening to historical subjects by various men, but as far as I am aware no one has considered medicine. With your consent, I will try to give you some of the curious phases of the practice of medicine in the past, connecting them with our own follies of the present.

Many present have attended lectures on medicine and other subjects, so let me read to you the manner of giving an anatomical lecture in 1581. This lectureship was founded by Lord Lumley and is in existence today. The stewards were ordered "to see and provide that there be every year a mat about the hearth in the hall that Mr. Doctor be made not to take cold upon his feet, nor other gentlemen that do come and mark the anatomy to learn knowledge. And, further, that there be two fine white rods appointed for the doctor to touch the body where it please him; and a wax candle to look into the body, and that there be always two aprons to be from the shoulder downward and two pairs of sleeves for his whole arm with tapes" The doctor was directed "to dissect openly in the reading place all the body of man, especially the inward parts, for five days together, as well before as after dinner, if the bodies may last so long without annoy." Between the lectures it was the custom for the audience to regale themselves and spiced wine was presented after each lecture. This thoughtfulness on the part of the benefactor in providing refreshments after each lecture is a custom which might be followed today, for often it is needed.

The great fire in London was looked upon as a catastrophe, which, no doubt, it was, but at the same time it cleaned the city as it never had been before, and saved it from future visits of the great plague. As conditions breed customs, let me quote

1. An address delivered before the Buffalo Historical Society, May 17, 1903.

from Erasmus and thus convey an idea of the filth in the houses. "The floors," he says in his letters, "are commonly of clay, strewed with rushes under which lie undisturbed an ancient collection of fleas, grease, fragments of bone, spittle, excrement of dogs and cats and everything that is nasty." If such was the condition of the well-to-do, what must have been that of the poorer classes? Smollett speaks of the sewage question in the Adventures of Peregrine Pickle, when at that time it was the custom to empty out of the window early in the morning the buckets in use during the night and to cry "look out." Unfortunately the warning was often uttered too late. Yet is it very different today in Baltimore where the sewage is open and where through the gutters runs unspeakable filth? Is there much difference between the floors of which Erasmus speaks and the omnipresent feather bed, "the bed which belonged to Grandmother," whose accumulated filth of deaths, sickness, diseases and births are carefully preserved and, added to from generation to generation, are handed down with such extraordinary care?

With such conditions as we have spoken of in England several hundred years ago, it is little wonder the plague and smallpox made such ravages. In such times when thousands were dying daily, irregular practioners sprang up all over the kingdom. We find in the *Oxford Journal* of February 11, 1758, the following advertisement:

I, George Ridler, near Stroud, in the County of Gloster, Broadweaver, at the desire of the people hereabout, do give nautis, that I have inokelated these two seasons past, between two or 300 for the smallpox, and but two or three of them died; a mainy people be afeared of the thing, but exaith it is no more than scrattin a bit of a haul in their yarm, a pushing in a piece of skraped rag dipt in some of the pocky matter of a child under the distemper. That every body in the nation may be served, I will, God willing, undertake to inokelate them with the pervizer that they will take the purges before hand, and lose a little blood away, for half a crown a head, and I will be bould to say, nobody goes beyond me.

N. B. Poor volk at a shilling a head, *but all must pay for the purging.*

As the disease was eradicated there must have been an overcrowding of the profession—a condition which is not unknown today—for we see in the want column of a paper of the period the following:

Wanted for a family who have had bad health, a sober, steady person, in the capacity of doctor, surgeon, apothecary, and man midwife. He must occasionally act as butler, and dress hair and wigs. He will be required sometimes to read prayers and to preach a sermon every Sunday. A good salary will be given.

This is almost as interesting as the *Herald* personals.

To those who have suffered, who have been subjected to all manner of cruel and disgusting practices, our sympathy should go. We do not appreciate now, those whom we might call the martyrs of medicine. They have certainly paved the way for the modern preparations of the pharmacist's art and surgical science. In the time of Henry VIII., the surgeon of the period dressed wounds with acid balsams, so that matter might form, which was considered necessary for a cure, every flap of skin being cut away. Wounds were seldom sewn, but if too small, were dilated with hideous forceps and torn open. In compound fractures, dressings were thrust between the ends of the bones so they would heal! Bullet wounds, considered poisonous from being inflicted by lead, were treated with applications of red-hot irons and boiling oil. It was only the result of an accident that caused simpler methods to be used. Ambrose Paré, who was the chief surgeon of the French for 25 years, found in one of the sieges of the army, that his supply of boiling oil had given out and he was obliged to use simpler applications, though not without serious apprehensions of finding his patients in horrible condition. To his astonishment he found those treated without the boiling oil were in far better condition than those treated with it, hence less barbarous methods were substituted.

This same Ambrose Paré wrote a book and as a modest man —as surgeons are apt to be—he wrote the following in the preface:

You must know that for the space of three years I have lived in the Hotel Dieu, of Paris, where I had the means of seeing and knowing *all* which can be of alteration and disease in the human body, and to learn from an infinite number of dead *all* that can be said of anatomy.

Paré was a good anatomist and by far the greatest surgeon of his time, the confidential friend of four successive kings, and is said to have been the only Protestant in Paris who was spared the massacre of St. Bartholomew's, a circumstance due to the direct action of the king. (Billings.) That the surgeon was well named,—the derivation being ergon, work, and cheiro, the hand—(we get the same derivation in "Cheiro" the palmist, who by the way learns much from our old friends, and who shows that we are still in the coils of superstition, omens and signs), the following account of one gentleman's method concerning amputations, would bear witness:

"First of all advise the patient to resign himself to God, to confess his sins, to remember the suffering of our Lord with thanks and the surgeon the same; thus will God grant him good fortune in his work."

Another account of an eye witness of an operation (diary of the Rev. John Ward, Vicar of Stratford-on-Avon, extending from 1648-1679): "A cancer in Mrs. Townsend's breast, of Alverson, taken off by two surgeons . . . First they cutt the skin cross and laid itt back, then they worked their hands in itt, one above and another below, and so till their hands mett, and so brought it out . . . There came out a great quantitie of waterish substance, as much as would fill a flagon. They put in a glass of wine and some lint, and so lett itt alone until the next day; then they opened itt again, and injected myrrhe, aloes and such things, as resisted putrefaction and then bound it up againe . . . The way how it should be cutt was markt with ink by one Dr. Edwards. Gill told me of a woman that had an apostheme about the side, and his master intended to trepan her on one of the ribs, whether it cannot be; I suspected itt to be a ly."

Guy de Chauliac, a French surgeon, flourished in the XIV. century, and in 1363 wrote a treatise on surgery, which he divided into five parts. Only one interests us,—namely, the fifth, from which I quote: "The fifth sect is of women and many fools, who refer the sick of all diseases to the saints, solely saying, "The Lord has given me that which it has pleased him. The Lord will show me what it pleases him. Let the name of the Lord be praised. Amen." This was written nearly six hundred years ago, but does it not hold true today of the misguided who travel to Lourdes and other shrines in search of health through religious intervention? Saint Anne de Beauprée was unknown at that time. From the fallacy of the wondrous power of healing of sacred objects and religious celebrities, it is easy to pass one step further down in the scale, and come to the wondrous power of healing by the weapon which produced the wound,—the sympathetic or magnetic cure of wounds. The cure was to anoint the bloody sword or other weapon which had inflicted the wound, or a stone or cloth dipped in the blood as it flowed from the wound, with a special ointment, and put it away carefully and apply nothing to the wound but some lint. (Billings.) This idea in a modified form is still existent today, as everyone is familiar with the superstition of killing the dog that has bitten a person to prevent madness. In some parts of the country the cure for a dog bite is to take several of the dog's hairs and smear them with blood from the wound, bury them, after which hydrophobia need not be feared.

But here is a learned gentleman, one Dr. Caius, who devoted a treatise to the medical properties of a well-known lap-dog, to which he gives the names of "Gentle Spaniel." He says:

These little dogs are good to assuage the sickness of the stomach, being often times thereunto applied as a plaister preservative, or borne in the bosom of the diseased and weak person, which effect is performed by their moderate heat. Moreover, the disease and sickness changeth his place and entereth—though it be not precisely marked,—into the dog which, to be no untruth, experience can testify. For this species of dog sometimes falls sick, and sometimes dies without any harm outwardly enforced.

That dogs sometimes die is sufficient proof that they are of service to the sick. Please be good enough to remember that this same Dr. Caius, is the one who was the founder of the College in Cambridge which bears his name.

The modern operation of skin grafting was overshadowed by an Italian whose name is revered by surgeons today, as being the inventor of a method of engrafting new noses on old faces. The necessity for such work was more pressing in those days as a frequent punishment was to cut off noses and ears. The graft for the new nose is obtained from the arm of the patient. When first proposed it was commonly supposed that the graft was obtained from the hindmost extremity of a well paid martyr, and so strong was the sympathy between the nose and the parent stock that when the martyr died, the nose fell off. The homeopathic idea—like cures like—is older than the name, for we find an Italian surgeon, one Phioravanti, in 1580, advising the following: "The reason why white of egg is to be used in mixing applications for wounds is because the white is that part which produces the flesh, the skin, and the feathers of the hen, while the yolk engendereth only the intestines. Therefore the white is like unto flesh, and its special business is to produce it." This idea of using the white of egg exactly for the same reason has come into vogue in the past thirty years, and has been used successfully in place of flakes of skin in skin grafting. That like cures like, we have a different phase put on the matter by some of the old Irish physicians, who adapted the color of medicines to the color of the disease. For example, a remedy for jaundice is a yellow medicine, as saffron, tumeric, sulphur and even yellow soap. Unfortunately for this theory we have not been able to find a cure for the blues.

It is interesting, in this relation, to recall the reason why barber poles are red, white, and blue. The design comes from the habit of bleeding, the white representing the fillet or band, the red the arterial, and the blue the venous blood. In the old days bleeding was very much in vogue and usually done by barbers. People were bled for indigestion, for almost every ailment, before taking a journey, at the end of the same, before making a will—a wise precaution on the part of the medical adviser—in fact any event of importance was often preceded by the important custom of bleeding. And in the XVIII. century in the hard drinking days of George the III., when full-bodied claret, port, and rum were consumed in enormous quantities, the practice had much to commend itself. Advertisements may be found in papers of 1701, that "at Hummins's, in Covent Garden, one might sweat in the cleanest and be cupped in the newest manner." A man named Radcliffe, in 1703, had an attack of pleurisy, which nearly cost him his life. Bernard, the sergeant-surgeon bled him to the amount of one hundred ounces, or six pounds weight of blood, and checked the disease. The poorer class of physicians used to make a practice of being present at the drawing of the prizes at the lotteries at Guildhall, in case the sudden announcement of winning would have an overpowering effect on the holder of the ticket. The charge for cupping was high,—5 shillings, 6 pence.

Thomas Dover, from whom we derive our familiar Dover's powder, was an ardent admirer and advocate of cupping. He thus described a cure of consumption in 1733:

One, Mr. William Masters, an eminent surgeon at Evesham, in Worcestershire, was so far gone in a consumption that he was not able to stand alone. I advised him by all means to lose six ounces of blood every day for a fortnight, *if he live so long;* then every other day, then every third day and fifth day for the same time. This was in the month of November. The March following, he rode from Evasham to Bristol in one day, which is forty-seven long miles, to give me thanks for his recovery. He lived many years after.

Dover was a curious character, living in Bristol, England, where he followed the occupation of physician and apothecary. When business was dull he would fit out a privateer and sail to the Spanish Main and replenish his empty purse, and no doubt found buccaneering more profitable and more exhilarating than medicine. To show the decrease in the practice of bleeding, we may say that in 1839 more than one million leeches were consumed in the Paris hospitals alone, while forty years afterward the annual supply was less than fifty thousand.

Medical enthusiasms are not new by any means. Let us see how mercury was administered in 1732. One of the most ardent champions of crude mercury is our friend, pirate, buccaneer, surgeon, apothecary, Thomas Dover. Crude mercury was a panacea, and many persons without consultations swallowed an ounce of mercury, twice a day for weeks. In a letter dated 1723, a gentleman who was a confirmed dyspeptic, wrote that after a course of treatment he found himself "in a pretty good state of health." An actor named Booth had jaundice and an intermittent fever, and he resolved to place himself under the care of Dover. I quote: "the doctor assured him that the crude mercury would not only prevent the return of his fever, but effectually cure him of all his complaints." There was no mistake in the prognosis. "On the 4th of May he began a mercurial course as directed "and by Tuesday the 8th of May (in four days time)" he had taken within two ounces, two pounds weight of crude mercury. He now began to complain of very great pain and general uneasiness under which he suffered. "He could not remain in the same posture one moment." His wife called in another physician who "ordered nine ounces of blood to be drawn from the jugular vein." In the evening "a cordial mixture" was administered—probably Sir Walter Raleigh's Cordial. The next day the headache still continuing, an epispastic was laid over the scalp and an emulsion prescribed. That night Mr. Booth died. This will afford a very good idea of the treatment in the early part of the XVII. century.

A large number of our English ancestors suffered greatly from apoplexy,—which we have shown was treated so well by bleeding—and from calculus or stone. A certain nostrum prepared by Mr. Stephen—it will be seen that Mrs. Winslow had her prototype—was largely used as a solvent. A certain Dr. Hartley was its most enthusiastic advocate and he published a number of pamphlets on its efficacy. Yet his endorsement and faith, alas! were shaken as he himself died of calculus after taking over two hundred pounds weight of soap, which was largely the base of the so-called solvent. That the inventors or originators of medical specifics often die from the same diseases which they guarantee to cure is well known. Lydia Pinkham is said to have died from the same diseases which we are assured she cured. A certain so-called cancer specialist in Buffalo, who professed to cure cancers, died from that disease.

One would think that our old gentlemen after diagnosing the condition by the color, and prescribing crude mercury by the pound, would be a bit chary about the prognosis. Not a bit of it, for we are told by the Irish practitioners how to foretell the termination of a fever: "Take a black cock, split him open and, while the halves are still hot apply the two halves to the patient. Should they stick on, the patient will recover, but should the pieces fall to the ground, death is certain." In certain parts of England today, when a child is born with a rupture, an ash tree is split and the child is passed through the opening. If the tree unites or heals, so will the rupture unite, hence the child will be well. But how slow and cumbersome a method to wait until trees grow. Listen to this. Culpepper says: "Number the days from the twenty-sixth of June, to the day when the party first began to fall sick, and divide the number by three; if one remain, he will be long sick; if two he will die; if none he will quickly recover."

We may laugh at this crude way of ascertaining the end of a sickness, but at that time possibly we could excuse such methods. Today there are a number of people who should know better and whose manner of living certainly justifies one in believing that they have some intelligence, yet they consult a humbug on East Eagle Street.

I learned the following from a patient who was shot in the chest. His wife not content with the hospital's treatment went down to see this female quack and came breathlessly back with a piece of old newspaper, on which was scrawled, "the bullet is between the lining of the lung and the covering of the heart." The ball was taken from his shoulder.

It has been said that dental surgery dates back and was made fashionable by royalty, as the Princess Mary—better known as Bloody Mary,—caught cold at the lying-in-state of Queen Jane, at Hampton Court Chapel, and the cold settling in her face necessitated the removal of one of her teeth. Should you have a toothache let me assist you in the Irish way: the tooth of a dead horse or the hand of a dead man rubbed into the jaw will be found efficacious, or should the whole side of the face ache, try this: "Drink water from a human skull or take a pinch of tobacco from the priest's pipe and put it in your mouth; then kneel down and say a Pater or an Ave and you will have no more toothache as long as you live." Christian science again. As many people today believe that the efficacy of medicine is in direct proportion to its evil taste or odor, so in the older days. Today in the South we often hear from the negro, "that's right powerful medicine, Doctor." Acting on this supposition we find remedies which would please the most exacting taste. Those who go to the apothecary's and chafe at the delay at the filling of prescriptions should consider yourselves fortunate that the physician did not prescribe "bees prepared in winter," or

"four or five ounces of peach kernals in the spring." At times a prescription called for parrots tongues, hawks livers, and "you need not stare," our informant writes, "if your bill amounts to pounds sterling." A very expensive remedy was unicorn's horn, which sold in the XVII. century for £24 sterling an ounce. A Dr. Hodges administered it to some patients afflicted with the plague, but did not get as satisfactory results as he did with troches of vipers, while troches made from the rattlesnake were more stimulating and better; and today rattlesnake oil is not an uncommon remedy in many regions.

Here is a cure for gout from a learned gentleman who flourished in 1659:

Take a young puppy, all one color-mark, if you can get such a one, and cut him through the back alive and lay one side hot to the given place, the inside I mean." Again, here are some Irish cures, this for whooping cough: "Put a live trout in the child's mouth, fasting. Then put it back alive in the stream. If a trout cannot be had, a frog may be tried." For mumps: "Tie a halter around the child's neck and lead him to a brook and bathe him, dipping him three times in the name of the Trinity." For foreign bodies here is a charm that has more benefits than the Rëntgen rays, as one always can use it. I quote from Amidenus who lived in the VI. century: "Bone, as Jesus Christ caused Lazarus to come out of the grave, as Jonah came out of the whale's belly, come out!"

A standard remedy, but one which was rather expensive, was the oil of kittens. This was prepared by boiling live kittens, coats and all, in olive oil. This subtle combination went by the name of oleum cattalorum and it took Ambroise Paré two years of hard work and a large sum of money to get the formula from a Florentine surgeon. Lest this provoke too much derision, I beg to call attention to the fact that a tincture which we use today called Warburg's, has as one of its principal ingredients dried bellies of skunks. Means to promote anesthesia was the ideal sought by all men for centuries and various methods were in use to lessen the sensibility to pain. In a book called "The Physicians of Middvai," published in the XIII. century, a chapter is devoted to producing anesthesia.

Take the juice of the poppy, orpine, eringo, mandrake, ground ivy, hemlock and lettuce, equal parts. Let clean earth be mixed with them and a potion prepared, then without doubt the patient will sleep. When you are prepared to operate upon the patient, direct that he shall avoid sleep as long as he can and then let some of the potion be poured into his nostrils, and he will sleep without fail. When you wish to awake him, let a sponge be pounded in vinegar and put in his nostrils. If you wish that he should not wake for four days, get a pennyweight of wax from a dog's ear and the same quantity of pitch; administer it to the patient and he will sleep.

The same gentleman who cured gout with puppies advises this for swellings:

Mark where a swine rubs himself, then cut off a piece of the wood, and rub any swollen place with it and it will help it; with this proviso, that where the hog rubs his head, it helps the swellings of the head, and where the neck, those of the neck. If you cannot apply a part of the thing the hog rubbed against, to the grievous place, you must apply the grieved place to that.

A quack of two hundred years back had a plaister good for green wounds, old fistulas, and ulcers, pains and aches in the head, limbs and elsewhere, as well as a sovereign remedy for contusions, tumours, king's evil, sprains, fractures, dislocations or hurts inflicted either by sword, cane, or gunshot, knife, saw or hatchet, hammer, nail or tender hook, fireblast or gunpowder. He also had a cordial, which while it strengthened the heart and promoted the appetite, proved also a sovereign remedy against worms, as well as a delightful dentrifice. While this was printed 200 years ago, yet we could name half a dozen advertisements with very similar wording and assurances to cure as diverse a number of diseases. The present advertisements of quack medicines all have testimonials attached, but they do not have the true ring when compared with these. I present a specimen in vogue at that period:

Trinity College, near Dublin, March 7, 1684.

Though the art, experience and reputation of Mr. William Read, practitioner of physic, chirurgy, and a great oculist, be sufficiently known in England and Scotland, where he has long exercised his skill with very good success; yet since he has but lately come into His Majesty's kingdom and has desired our testimonial concerning his performances here, we do certify, that he has done several remarkable cures with great dexterity and success; as the couching of cataracts, cutting off cancerated breasts, mortified arms and legs (and very little effusion of blood, by virtue of his excellent styptic water), several of which operations we have, with very much satisfaction, ourselves seen him perform, as we do testify under our hands and seals, the day and year above written.

Signed: Narcissus (Lord Bishop of Ferns) and Leighton; Robert Huntington, Provost; Allen Mullin, M.D.

This same William Read was knighted by Queen Anne in token of his marvelous work in medicine. That there have been hundreds of scalawags of the Antonius order, we find this delightful reminder:

As it would be no less disrespectful than injurious to the public to conceal the merits of Mr. Grant, oculist, therefore we, the minister, churchwardens, and overseers of the Parish of St. Mary, Newington Butts, do hereby certify, that William Jones of the said parish, aged twenty years, who was born blind, on his application to Mr. Grant the aforesaid, who dwells in the St. Christopher's Court, behind the Royal Exchange, by the Blessing of God, in the skilful hand of Mr. Grant, the said Jones, in five minutes time, was brought to see, and at this time, hath his sight very well. This case being so particularly remarkable, and gratisly performed, we do, therefore, give this public testimony, under our hands, this 25th of July, 1709.

| | |
|---|---|
| Minister, | WILLIAM TASWELL. |
| Churchwardens, | JAMES COMBER, |
| | WILLIAM DALE. |
| Overseers, | FRANCIS TROSSE, |
| | WILLIAM BENSKIN, |
| | WALKER WOOD, |
| | JOHN SHIP. |

Charms have always occupied a prominent place in the cure of disease from time immemorial. A ring made from the hinge of a coffin had the power to relieve cramps, which were also cured when a rusty sword was hung by the patient's bedside. To cure toothache, in addition to the many I have reported already, a nail driven into an oak tree was a preventive. A halter which had served to hang a criminal, when bound around the temples, was an infallible remedy for headache. The moss growing on a human skull, when dried and pulverised and taken as a snuff was another headache cure. A dead man's hand could dispel tumors of the glands by stroking the parts nine times; but the hand of a man who had been cut down from a gallow's tree was infinitely more efficacious. To cure warts, the best thing was to steal a piece of meat from the butcher, with which the warts were rubbed and the meat buried. As the process of decomposition went on, the warts would wither and disappear. The chips of the gallows on which several persons had been hanged were an excellent cure for the ague. The nightmare, which was supposed to be caused by supernatural agency, was banished by means of a stone with a hole in it, being suspended at the head of the sufferer's bed. This last named remedy went by the name of "hagstone," because "it prevented the witches, who, of course, brought the mischief, from sitting on the patient's stomach." (Everitt.) I know of several men who carry horse chestnuts in their pockets to ward off rheumatism.

This interesting bit of information, clipped from a recent edition of the *Buffalo Express,* will be appreciated, I am sure:

MADSTONE MAKES A RECORD.

Newcastle (Ind.) corr. *Indianapolis News.*

The Bundy madstone has just completed the longest record of clinging known in its history. The case was that of Mrs. A. Silcox of Attica, O., who was bitten by a dog last January. The stone clung to the wound for 234 hours. Beside drawing out the poison caused by the bite, it drew from the wound a quantity of arsenic administered by the physicians to counteract hydrophobia. Homer Bell of Muncie is now under treatment and three cases are in waiting.

The power of rings to cure diseases of the mind, body, and heart comes to us with a most venerable pedigree. To endow them with peculiar virtue elaborate ceremonies had to be observed. The mode in which the rings received their peculiar healing virtues is described in *An Ancient Book of Ceremonials of the Kings of England.* The ceremony seems to have been usually performed on Good Friday, or some other day set apart for devotional purpose, we are told.

The King would come to his chapel or closet with his lords, without any sword being borne before him, as was usual in other public ceremonies, and there he waited until the bishop and dean had brought the crucifixe out of the vestrie and laide it upon the cushion before the High Altar. It was then the duty of the ussher to lay a carpet for the Kinge to creepe to the crosse upon, and that done a form was sett upon the carpett before the crucifixe and a cushion laid upon it, for the Kinge to kneel upon, and the Master of the Jewell House was to stand ready with the crampe-rings in a basin of silver, while the clerk of the closet also waited with the book concerning the hallowing of the crampe rings, the almoner at the same time kneeling on the right hand of the Kinge, holding the sayd booke. When that is done, the Kinge shall rise and go to the Altar, wheare a gentleman ussher shall be redie with a cushion for the Kinge to kneel upon. And then the greatest of Lords that shal be ther to take the basin with the rings, and beare them after the Kinge, to offer. (Everitt.)

Surely with such ceremony the rings should have miraculous power. How many persons today are wearing rheumatic rings? How many declare that since wearing such rings they have been free from pain? A commoner example of the efficacy, which rings have in the mind of people, is the ordinary marriage ring.

Why was such an emblem adopted, signifying all that it does, were it not that the wearing of rings was primarily based upon superstition and charm?

If time permitted it might prove of interest to give some idea of the importance with which spitting was looked upon and how it has been brought down to various ceremonies and functions of today. I may say that in former times spittle was used in baptism and in many of the daily transactions of life, either as a lucky or confirmatory act. Even today, in Ireland, no bargain is concluded at a fair or market till the seller lays the money in his hand and spits on it. It has been employed in medicine and for a perfect result, the saliva should be used after a fasting, especially after a black fast, when the person has not even taken water. It is effectual for chapped lips, when mixed with dust and applied in the name of the Trinity. 'Tis a specific for warts, as I can testify to! For ague if one spits on a spider,—a small living spider,—then rolls it in a cobweb and puts in a lump of butter and swallows it, it cannot be surpassed.

Having told how to treat the various diseases and given many hints on prognosis and medicines, some information to those members of the medical profession who are here, no doubt, will be of interest. I will tell how to acquire a practice. I can do no better than to quote from an author in the early part of last century:

To visit the opera frequently, and be careful to instruct the messengers, when the performance was over, to loudly vociferate for your carriage. This is an effectual way of making you known as a London physician and a man of fashion. Be regular in your attendance at church; and instruct your servant to call you out occasionally during the service when you first start in practice. It will be of service if you can persuade your carriage friends to call often at your house. Always contrive to have a coach standing at your door on Sunday, as it is sure to attract the notice of the people as they return from church, and lead the public to believe that you are a practising physician.

The practice of being called from the theatre is as old as can be and seems to have been first put in vogue by a certain Dr. Kennedy of Nassua Street, Soho, who flourished toward the close of the XVII. century. I quote from Everitt:

Three nights of the week, at least during the playgoing season, the doctor was to be seen in the boxes of the Drury Lane or Covent Garden, and the doctor though an ostensible spectator was, himself, the principal performer in a farce of his own invention, in which the performers were three in number,—himself and two hired retainers. One of the latter would present himself at the house at which he knew the doctor was not present and dressed in a smart livery, he would call loudly for "Dr. Kennedy." The other rascal would then rush into the theatre at which the physician was present and bawl the name of "Dr. Kennedy" in stentorian tones. It was part of the performance that the doctor should then rise, and taking his hat and professional cane, depart bowing apologetically right and left, as he quitted the house. The performance though frequently repeated, drew attention and admiration. "Bless me," the great people would soliloquize, as the doctor intended that they should, "that Dr. Kennedy would seem to have half the patients in the town."

Do you not know some Dr. Kennedys in Buffalo?

It would seem from what has been said that we are not so far advanced after all, with our feather beds, our superstition about killing dogs that bite us, our Warburg tinctures with dried bellies of skunks, our rheumatic rings, our habit of carrying horse chestnuts in our pockets to ward off disease, and lastly, that pernicious fad, which is the outgrowth of charms, and amulets, Christian Science.

481 Franklin Street.

